# Staged Achilles Allograft Reconstruction of Chronic Bilateral Simultaneous Tears of the Retracted Distal Biceps Tendon Using a Novel Fixation Technique

**DOI:** 10.7759/cureus.25172

**Published:** 2022-05-20

**Authors:** Sreenivasulu Metikala, Brandon Portnoff, Paul Herickhoff

**Affiliations:** 1 Orthopaedics, Virginia Commonwealth University Health System, Richmond, USA; 2 Orthopaedics, Penn State College of Medicine, University Park, USA; 3 Orthopaedic Surgery, Penn State Health Milton S. Hershey Medical Center, State College, USA

**Keywords:** achilles allograft reconstruction, staged reconstruction, allograft reconstruction, tendon retraction, simultaneous bilateral distal biceps rupture, bilateral distal biceps rupture, cortical button, distal biceps rupture, chronic distal biceps rupture

## Abstract

Bilateral simultaneous rupture of distal biceps tendons is an extremely rare clinical entity that can result in significant morbidity for an active person if not addressed appropriately. Treatment becomes more complicated in a delayed presentation as the tendon retracts and scars to the adjacent tissues, thus precluding a primary tendon-to-bone repair. The present study is a case report of an active male with a two-month-old simultaneous rupture of both distal biceps tendons managed by Achilles allograft reconstruction and double cortical-button fixation technique that provided a satisfactory functional outcome.

## Introduction

Chronic distal biceps tendon ruptures are often associated with considerable retraction of the muscle-tendon unit, scarring, and tissue atrophy. Surgical reconstruction, in such situations, is not only technically demanding but also associated with higher complication rates [1,2]. Direct end-to-end repair may still be possible but requires a higher elbow flexion angle, which may result in postoperative failure or flexion contracture [3,4]. Non-anatomic tenodesis to the brachialis muscle is an alternative procedure, but the patient loses greater than 50% supination strength [5]. Chronic situations, therefore, frequently require tissue reconstruction using either autograft or allograft to bridge the tendon-to-bone defect [6-12]. This report is about a patient who presented two months after sustaining simultaneous bilateral distal biceps tendon ruptures while performing pull-up exercises. We describe the surgical approach of staged reconstruction using an Achilles tendon allograft with a novel fixation technique to the radial tuberosity and report early outcomes.

## Case presentation

**Figure 1:**
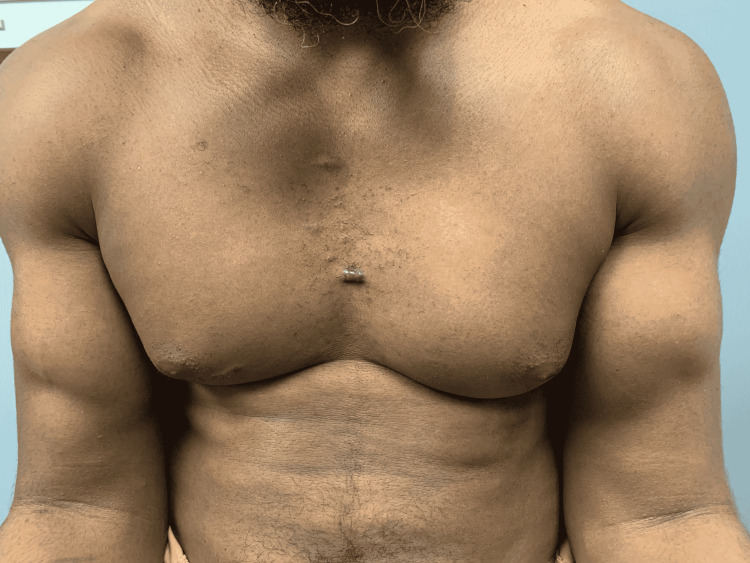
Bilateral distal biceps rupture with proximal retraction of muscle bellies

The patient was a 39-year-old healthy male inmate who presented to our orthopaedic service with a two-month history of bilateral elbow pain and weakness. The symptoms began during pull-up exercises with sudden “pop” in both elbows accompanied by immediate pain and cramping. He could not seek immediate medical attention due to the coronavirus pandemic causing a “lockdown” at his correction facility. He was a right-hand-dominant, avid weightlifter and denied any previous elbow pain, injury, substance abuse, or anabolic steroid intake. On physical examination, there was a noticeable ‘reverse Popeye’ deformity on both sides with significant retraction of the biceps muscle belly to the mid-arm level (Figure 1).

**Figure 2:**
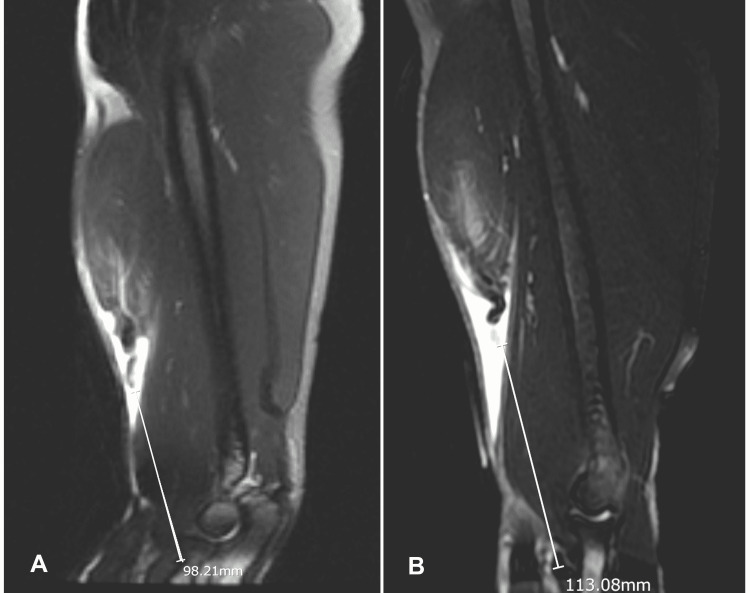
Sagittal T2 MRI images of right (A) and left (B) humerus Chronic full-thickness rupture of the distal biceps tendon with a gap of about 10 cm on the right (A) and 11 cm on the left (B).

On physical examination, the patient had pain on deep palpation with no ‘cord-like’ structure felt in the antecubital fossa, indicating a positive ‘Hook test’ [13]. He had reduced biceps strength with 4/5 flexion-supination power. There was a full range of elbow motion and forearm rotations. Clinical testing of the sensory and motor peripheral nerve function was unremarkable. The radial and ulnar artery pulses were both palpable. The above findings were similar on both sides. Anteroposterior and lateral radiographs did not demonstrate any osseous abnormalities. The right and left humerus magnetic resonance imaging (MRI) scans revealed complete tears of the distal biceps tendon, with 10 and 11 cm of retraction, respectively (Figure 2).

After a thorough discussion of the treatment options and postoperative rehabilitation, the patient elected to pursue operative management in a staged manner, starting with the dominant right side.

Operative technique

**Figure 3:**
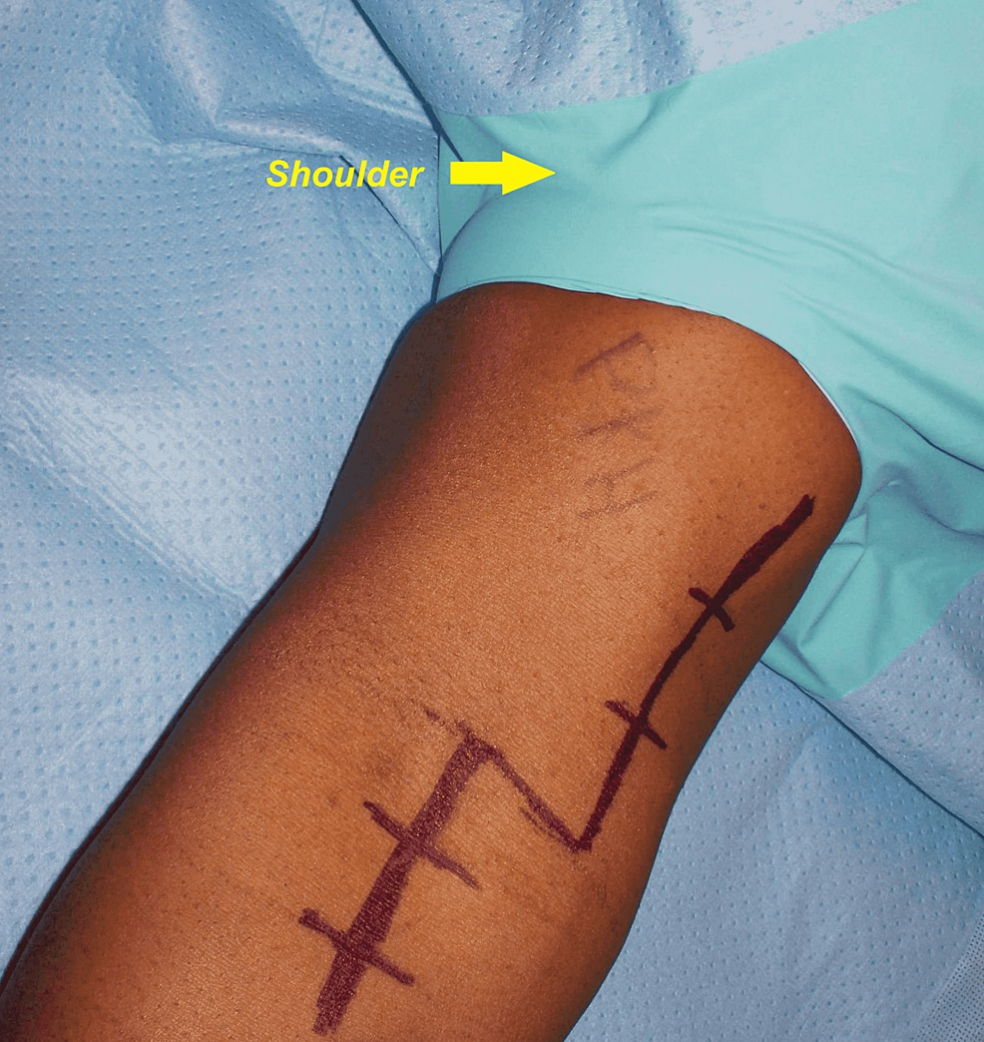
Henry's approach through a Z-incision for the right distal biceps reconstruction

The patient was placed supine with the right upper extremity positioned on a hand table. General anaesthesia was administered, followed by prophylactic intravenous antibiotics. A non-sterile tourniquet was placed as proximal as possible in the arm, adjacent to the axilla. After sterile preparation and draping, a multidisciplinary timeout was called. The extremity was exsanguinated, and the tourniquet was inflated to 250 mm Hg for 104 minutes. A long Z-shaped incision for a Henry approach was made, utilizing a transverse limb at the elbow crease with vertical extension proximally along the medial arm and distally staying medial to the mobile wad of Henry (Figure 3).

**Figure 4:**
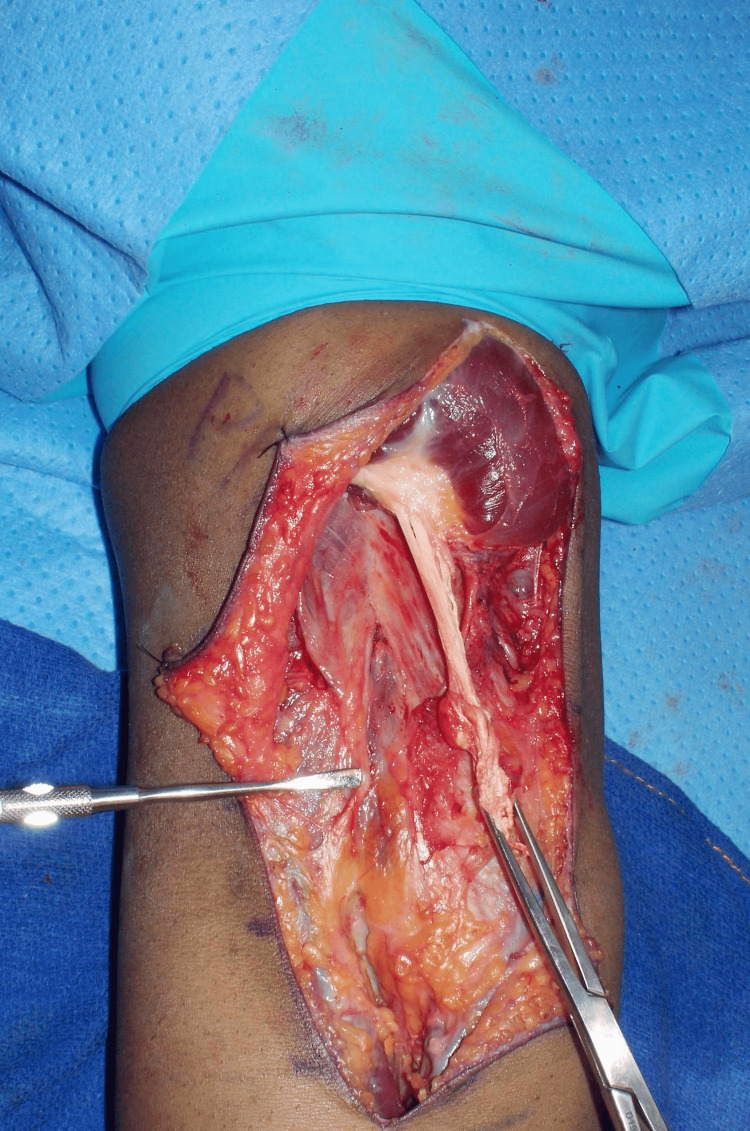
Deep exposure Scarred tendinous portion of the right distal biceps held with an Allis clamp. A Freer elevator is shown pointing to the lateral antebrachial cutaneous nerve.

Care was taken to protect the lateral antebrachial cutaneous nerve (LABCN) in the subcutaneous plane. Full-thickness flaps were raised on both sides and sutured to the adjacent skin with 3-0 nylon sutures. The lacertus fibrosus was found to be ruptured and contracted. Proximal dissection identified the scarred and retracted distal biceps stump (Figure 4).

**Figure 5:**
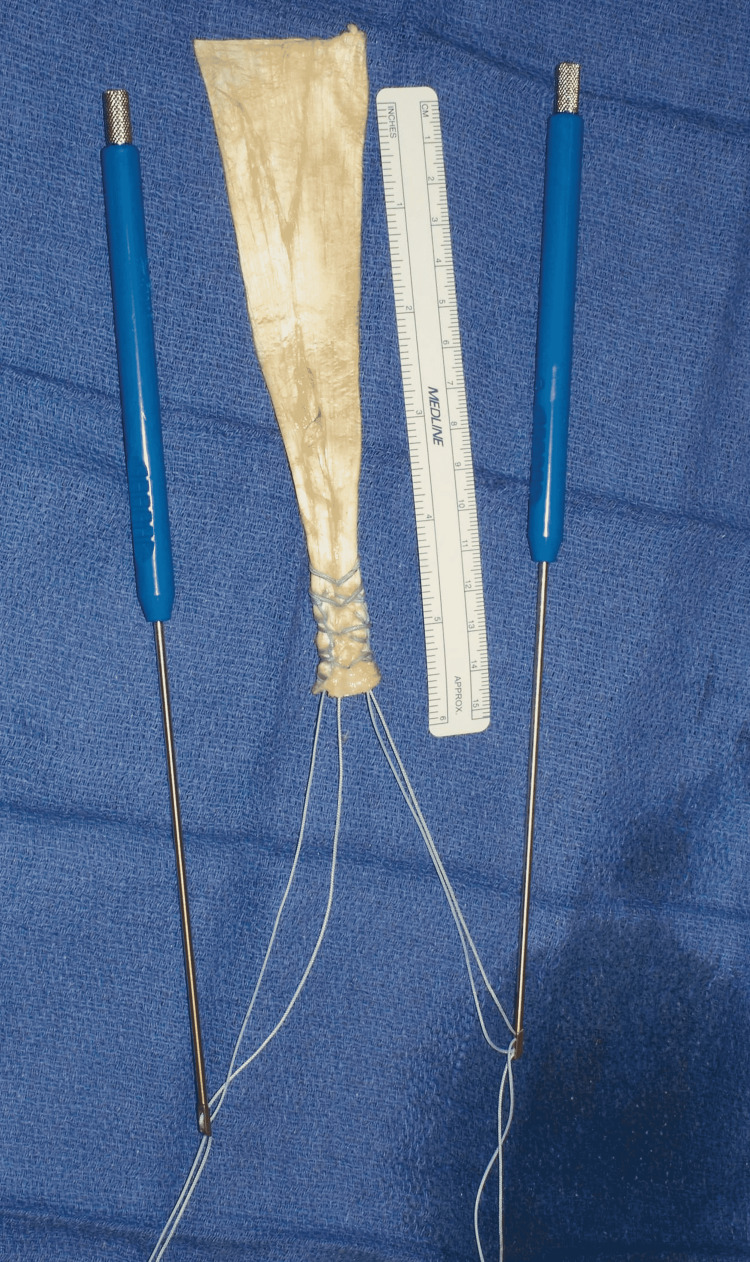
Prepared Achilles allograft held by two cortical buttons attached to insertion handles

The myotendinous unit was mobilized circumferentially by blunt finger dissection. The stump end was found to have degenerated and so debrided back to a normal appearance. Distally, the recurrent radial vessels were divided between suture ligatures, and the radial tuberosity was exposed. A trial reduction confirmed that greater than 90° elbow flexion was necessary for the direct tendon-to-bone reattachment. Therefore, the decision was made to proceed with an Achilles tendon allograft bridge reconstruction. The fresh-frozen graft supplied by the Musculoskeletal Transplant Foundation (NJ, USA) was utilized. The calcaneal bone block was excised, and the Achilles tendon portion was prepared on the back table. Using #2 FiberWire, the distal 3 cm of the tendon stump was secured on each side with four Krackow locking stitches, followed by a #2 FiberLoop whipstitch (Arthrex, Inc., FL, USA). Next, each pair of suture strands were threaded separately through two cortical buttons (Arthrex) and kept ready for insertion (Figure 5).

**Figure 6:**
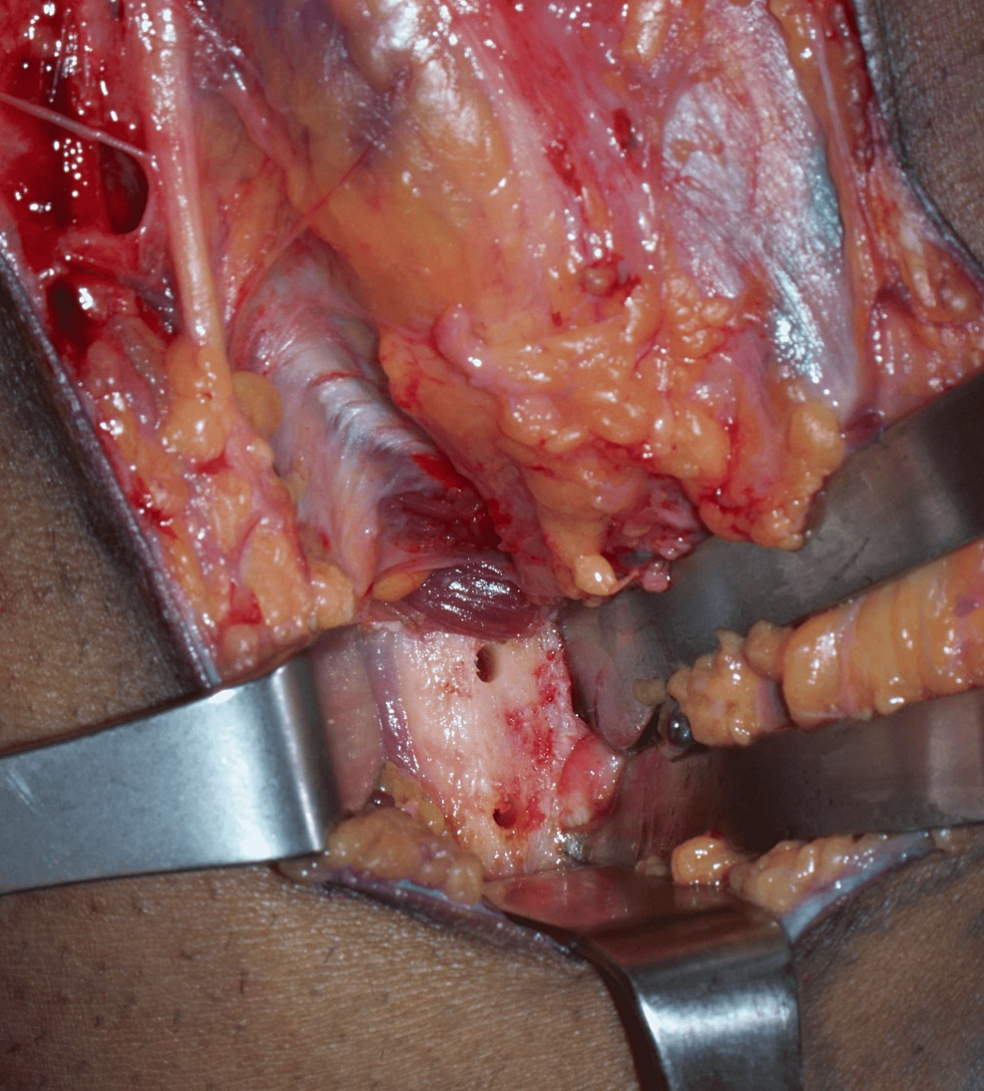
Two 3.2-mm unicortical drill holes at the radial tuberosity footprint of the right elbow

Attention was now turned to preparing the bony bed. The radial tuberosity footprint was identified by placing the forearm in maximum supination. The cortex was prepared using a combination of curettes and rongeurs to a bleeding surface. A 3.2-mm spade-tipped drill was utilized to create two unicortical holes, 1.5 cm apart, in a proximal-distal fashion into the ulnar aspect of the radial tuberosity (Figure 6).

**Figure 7:**
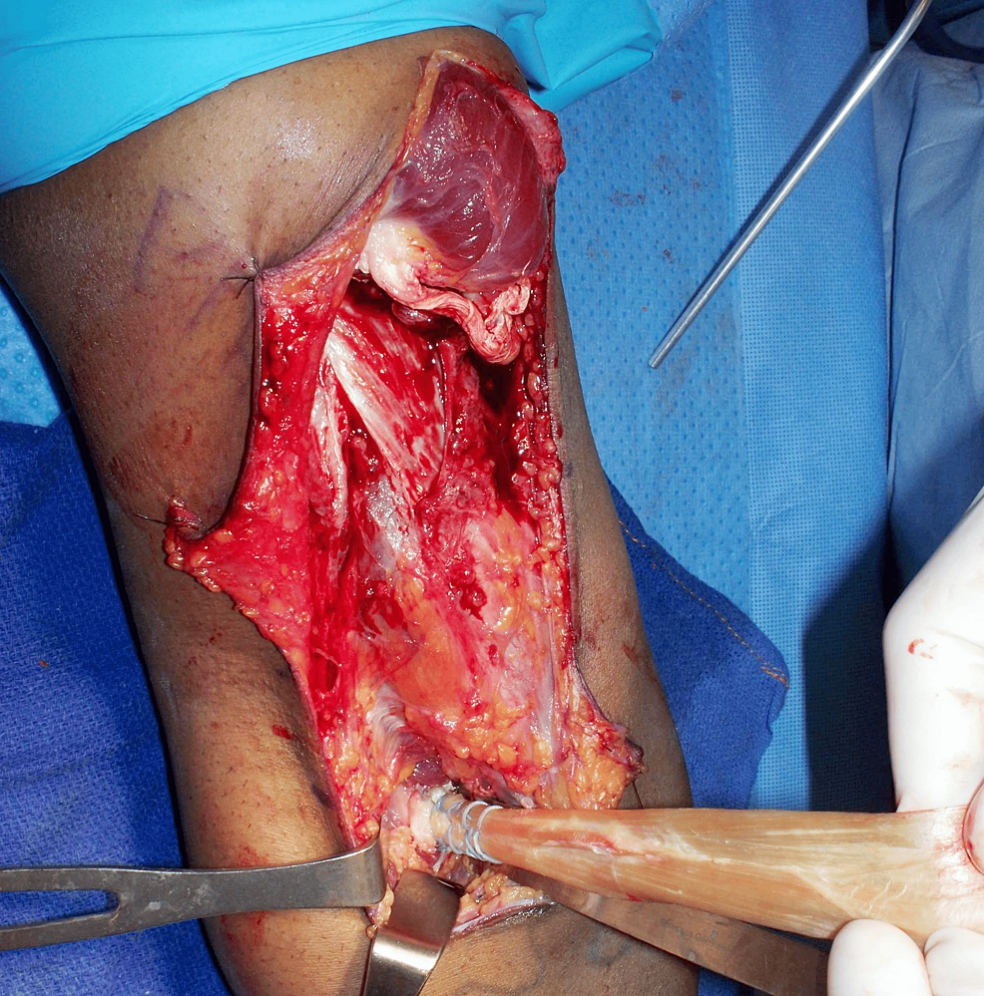
Distal attachment of the Achilles allograft to the radial tuberosity footprint of the right elbow

The wound was then irrigated with copious normal saline to remove bone debris. The prepared Achilles tendon allograft was brought into the surgical field, and the two cortical buttons were passed through the two cortical holes and flipped inside the intramedullary canal. The allograft tendon stump was reduced firmly onto the raw surface of the radial tuberosity footprint using a standard tension-slide technique, and the sutures were then passed back up through the tendon with a free needle and tied to one another, completing the distal fixation of the graft (Figure 7).

**Figure 8:**
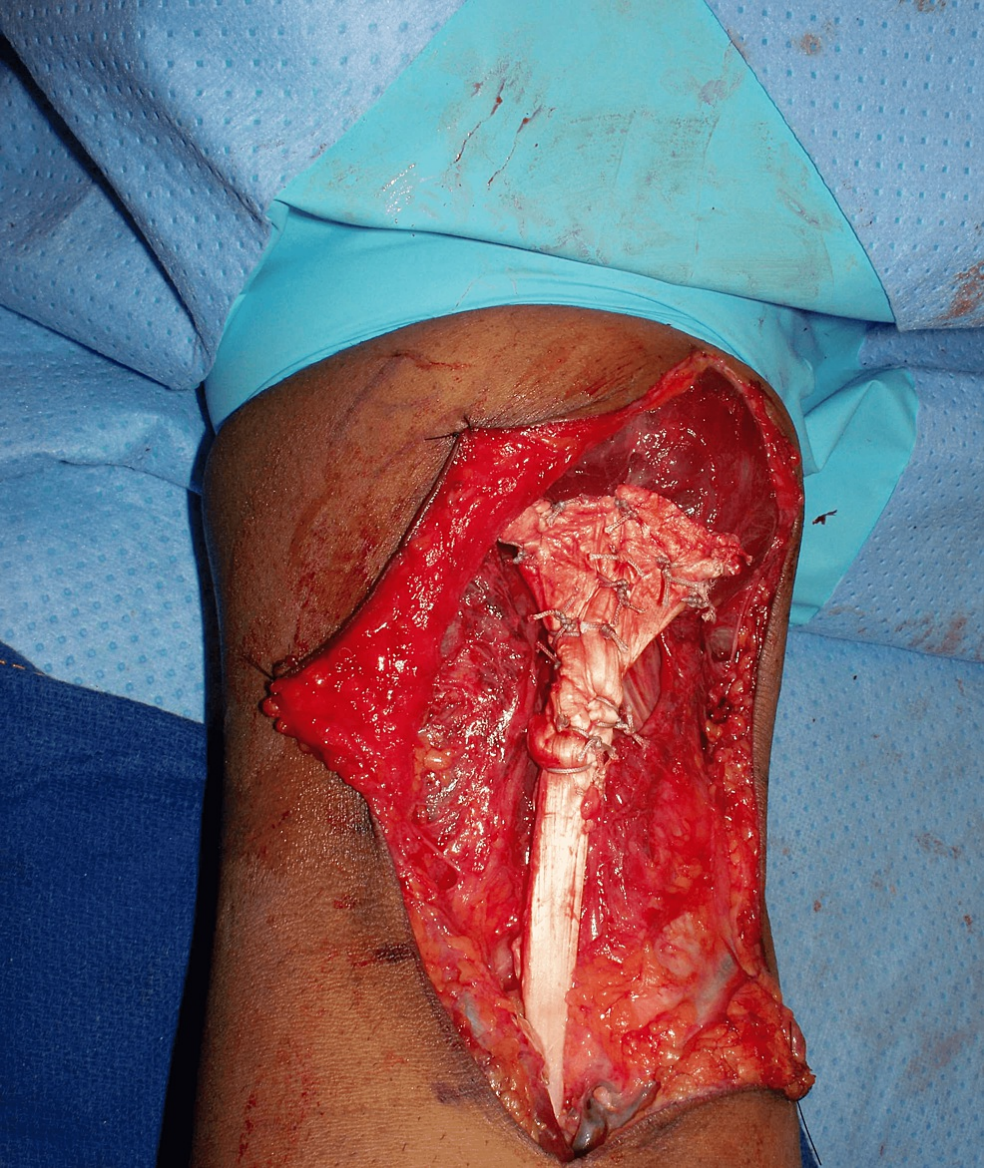
Proximal attachment of the Achilles allograft to the right biceps musculotendinous unit.

Attention was now turned to securing the allograft proximally. The broad tendinous part of the allograft was draped around the native biceps myotendinous unit. A longitudinal slit was made in the central portion of the graft material through which the biceps stump was pulled out to dial the tension for subsequent repair. Care was taken to position the elbow at 45° flexion to set the desired tension before securing with #2 FiberWire sutures. The remainder of the graft was then wrapped circumferentially and secured with multiple interrupted #2 FiberWire sutures, essentially tubularizing the native biceps with the allograft tissue (Figure 8).

**Figure 9:**
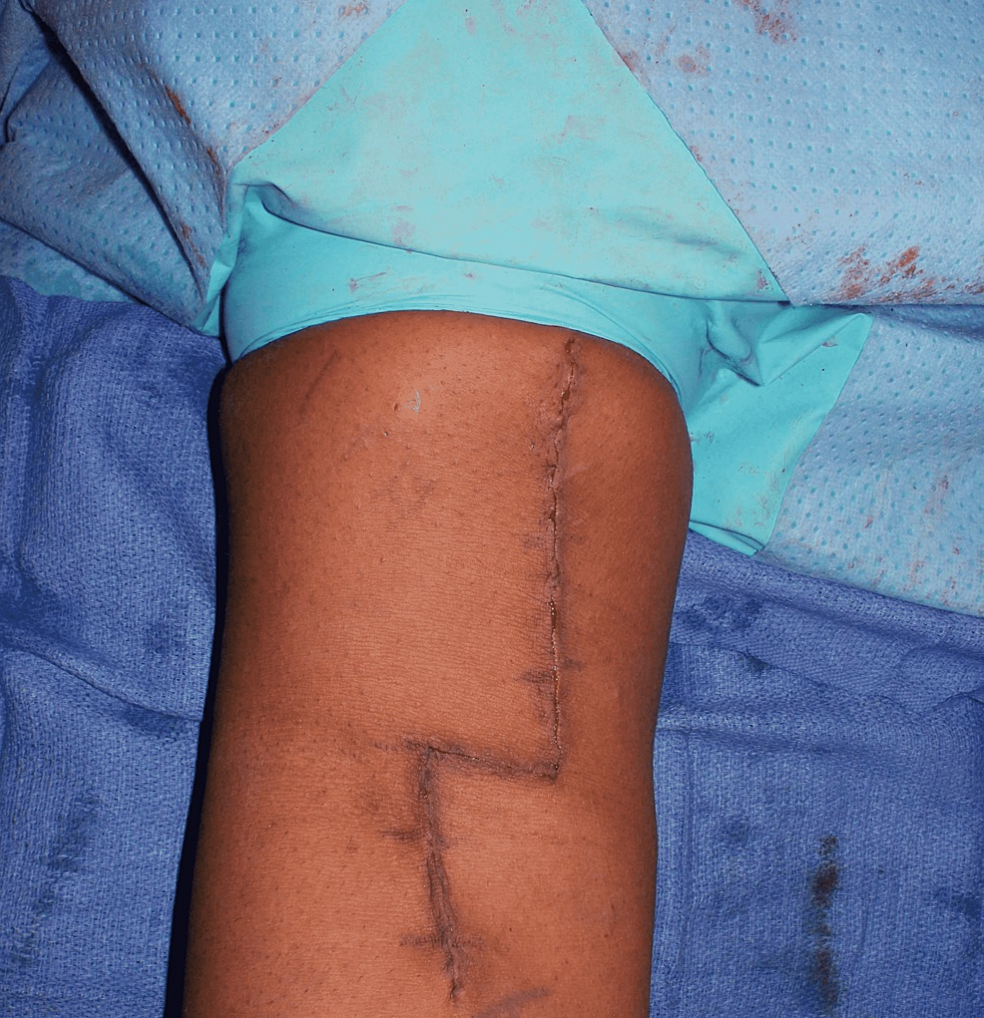
Subcuticular skin closure of the right distal biceps reconstruction

Finally, the reconstruction stability was tested by gentle elbow motion, and the surgical wound was closed in a layered fashion (Figure 9).

A posterior long-arm splint was applied over sterile dressings with the elbow in 70° flexion and the forearm in neutral rotation. The patient was discharged to his correction facility on the same day on oral pain medication. For the first four weeks, oral aspirin 81 mg, one tablet twice a day, was advised as deep vein thrombosis (DVT) prophylaxis.

Postoperative course and follow-up

The patient was then seen in the office two weeks post-surgery. The splint was removed, and the arm was placed in a sling to begin range of motion (ROM) exercises. A home-based rehabilitation program was started, including passive motion at the elbow and forearm, with the goal of achieving a full ROM over the next four weeks. At his next visit, six weeks post-surgery, the sling was discontinued to allow active ROM. At eight weeks, the patient started gradual resistance training to be continued until complete recovery. The Mayo Elbow Performance Index (MEPI) was utilized to assess the clinical outcomes [14]. Surgery to reconstruct the contralateral (left) side was performed at this eight-week mark, which was approximately four months post-injury, implementing the same surgical technique and postoperative course.

**Figure 10:**
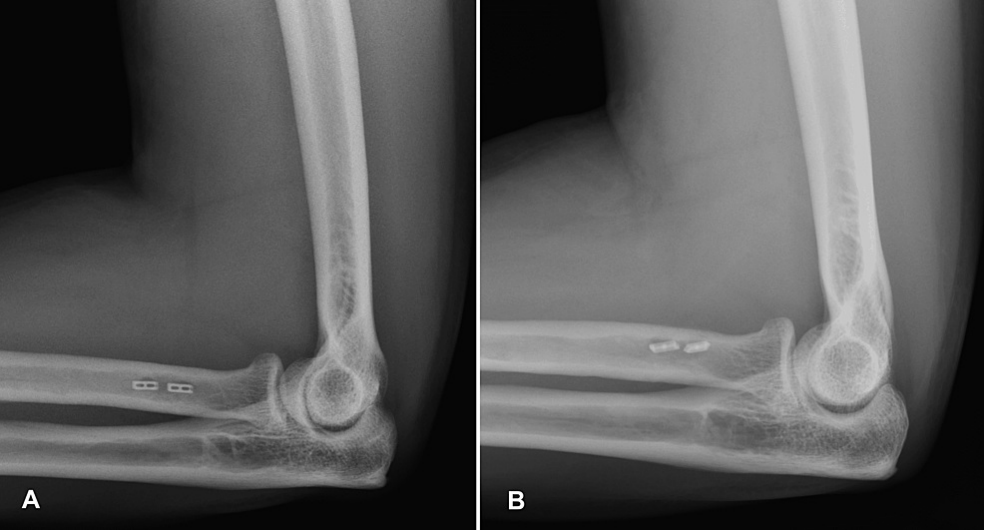
Postoperative lateral radiographs of the right (A) and left (B) elbows at the six-week follow-up showing stable hardware position

The incision sites were healed uneventfully, and no nerve-related complications were noted on either side. Plain radiographs six weeks after surgery showed the unicortical buttons to be appropriately situated in the intramedullary canal of the proximal radius, with no evidence of heterotopic bone formation (Figure 10).

The patient was unable to complete all the scheduled visits. He was lost to follow-up after the sixth-week postoperative visit for the left elbow (14 weeks for the right). The clinical examination at the last visit demonstrated full ROM with 5/5 biceps strength on the right side, whereas the left side had a 30° loss of terminal extension. No further follow-up visits could be scheduled, unfortunately, despite attempts to contact his correction facility. A summary of the results is depicted in Table 1.

**Table 1:** Summary of postoperative final clinical outcomes MEPI: Mayo Elbow Performance Index

	Right		Left	
	Pre-op	Post-op (14 weeks)	Pre-op	Post-op (6 weeks)
MEPI	70	100	70	85
Elbow flexion	135	135	135	135
Elbow extension	0	0	0	30
Pronation	90	90	90	90
Supination	80	80	80	80
Biceps strength	4/5	5/5	4/5	Not tested

## Discussion

With chronic bilateral simultaneous distal biceps tendon ruptures being a complex and rare condition, the literature is limited to isolated case reports. The present study describes the staged approach of surgical reconstruction using an Achilles allograft in a step-wise fashion secured by a double cortical-button fixation technique. The dominant right arm was operated at two months post-injury, followed by the left side at about four months, allowing the use of one upper extremity for his daily activities.

In general, a distal biceps rupture presenting more than four weeks from the initial injury is classified as a chronic rupture. It is subdivided into two categories based on the integrity of lacertus fibrosis [4,15]. A late primary repair is often possible without any graft if the lacertus fibrosus is intact, preventing proximal retraction of the torn tendon. However, it is not uncommon for the lacertus fibrosus to tear together with the distal biceps tendon. The muscle-tendon unit then continues to retract in a proximal direction in a matter of weeks, creating a substantial gap between the end of the stump and the radial tuberosity footprint. Furthermore, the retracted stump adheres to the underlying brachialis and adjacent intermuscular septum resulting in scar formation and atrophy. Due to the aforementioned reasons, the management of a chronic rupture is technically challenging and frequently requires some form of tissue augmentation to bridge the tendon-bone defect. Several autograft materials such as semitendinosus, fascia lata, palmaris longus, quadriceps, and flexor carpi radialis have been described with promising results [9,10,16]. Still, the possible donor site morbidity to reconstruct a bilateral situation, similar to the case reported here, can be a significant concern. Allograft reconstruction using an Achilles tendon has been recognized as an effective technique for the late reconstruction of the chronic distal biceps rupture [4,7,17,18]. Compared to others, the Achilles tendon allograft provides abundant tissue for a robust repair to the broad muscle-tendon unit. The technique of weaving the native biceps stump through the Achilles allograft tissue followed by tubularization of its broad aponeurosis offered multiple suture fixation points, enhancing the stability of the repair. Nonetheless, an allograft tissue takes longer to incorporate than an autograft material. Additionally, like with any allograft, disease transmission is a potential concern, which was discussed with our patient during preoperative evaluation.

With regard to the tendon-to-bone fixation technique, a cortical-button fixation has been shown to be associated with greater mechanical strength and load to failure than other methods [19]. Camp et al. described the surgical fixation of the acute distal biceps tendon rupture using a single intramedullary cortical-button with the advantages of minimizing the amount of native bone sacrifice and absence of violation of the far cortex, thereby reducing the risk of fracture [20]. To more anatomically reproduce the distal biceps footprint and provide a second point of fixation for the Achilles allograft, we elected to use two unicortical buttons spread apart by 1.5 cm. However, it should be noted that no biomechanical or clinical data exists to evaluate the superiority of a single intramedullary button versus two buttons. Our study is mainly limited by a short follow-up period of only four months. Further studies may be warranted to evaluate the long-term results of the Achilles tendon allograft reconstruction in the management of chronic bilateral distal biceps tendon ruptures.

## Conclusions

Late presentation of a bilateral simultaneous distal biceps tendon rupture is a challenging situation with limited surgical options. An Achilles tendon allograft, because of its abundant surface area for healing and ability to obtain secure fixation both distally and proximally, is a viable option for surgical reconstruction. Staged intervention is a reasonable approach as it allows the use of one arm for basic daily activities. The described fixation technique using two cortical buttons allows the surgeon to recreate the normal distal biceps tendon footprint for an overall satisfactory outcome.

## References

[1] Cohen MS (2008). Complications of distal biceps tendon repairs. Sports Med Arthrosc Rev.

[2] Litowski ML, Purnell J, Hildebrand KA, Bois AJ (2021). Surgical outcomes and complications following distal biceps tendon reconstruction: a systematic review and meta-analysis. JSES Int.

[3] Morrey ME, Abdel MP, Sanchez-Sotelo J, Morrey BF (2014). Primary repair of retracted distal biceps tendon ruptures in extreme flexion. J Shoulder Elbow Surg.

[4] Snir N, Hamula M, Wolfson T, Meislin R, Strauss EJ, Jazrawi LM (2013). Clinical outcomes after chronic distal biceps reconstruction with allografts. Am J Sports Med.

[5] Klonz A, Loitz D, Wöhler P, Reilmann H (2003). Rupture of the distal biceps brachii tendon: isokinetic power analysis and complications after anatomic reinsertion compared with fixation to the brachialis muscle. J Shoulder Elbow Surg.

[6] Zeman CA, Mueller JD, Sanderson BR, Gluck JS (2020). Chronic distal biceps avulsion treated with suture button. J Shoulder Elbow Surg.

[7] Darlis NA, Sotereanos DG (2006). Distal biceps tendon reconstruction in chronic ruptures. J Shoulder Elbow Surg.

[8] Dillon MT, Bollier MJ, King JC (2011). Repair of acute and chronic distal biceps tendon ruptures using the EndoButton. Hand (N Y).

[9] Hallam P, Bain GI (2004). Repair of chronic distal biceps tendon ruptures using autologous hamstring graft and the Endobutton. J Shoulder Elbow Surg.

[10] Levy HJ, Mashoof AA, Morgan D (2000). Repair of chronic ruptures of the distal biceps tendon using flexor carpi radialis tendon graft. Am J Sports Med.

[11] Sanchez-Sotelo J, Morrey BF, Adams RA, O'Driscoll SW (2002). Reconstruction of chronic ruptures of the distal biceps tendon with use of an achilles tendon allograft. J Bone Joint Surg Am.

[12] Wiley WB, Noble JS, Dulaney TD, Bell RH, Noble DD (2006). Late reconstruction of chronic distal biceps tendon ruptures with a semitendinosus autograft technique. J Shoulder Elbow Surg.

[13] O'Driscoll SW, Goncalves LB, Dietz P (2007). The Hook test for distal biceps tendon avulsion. Am J Sports Med.

[14] Longo UG, Franceschi F, Loppini M, Maffulli N, Denaro V (2008). Rating systems for evaluation of the elbow. Br Med Bull.

[15] Ramsey ML (1999). Distal biceps tendon injuries: diagnosis and management. J Am Acad Orthop Surg.

[16] Hang DW, Bach BR Jr, Bojchuk J (1996). Repair of chronic distal biceps brachii tendon rupture using free autogenous semitendinosus tendon. Clin Orthop Relat Res.

[17] Patterson RW, Sharma J, Lawton JN, Evans PJ (2009). Distal biceps tendon reconstruction with tendoachilles allograft: a modification of the endobutton technique utilizing an ACL reconstruction system. J Hand Surg Am.

[18] Phadnis J, Flannery O, Watts AC (2016). Distal biceps reconstruction using an Achilles tendon allograft, transosseous EndoButton, and Pulvertaft weave with tendon wrap technique for retracted, irreparable distal biceps ruptures. J Shoulder Elbow Surg.

[19] Taylor AL, Bansal A, Shi BY, Best MJ, Huish EG Jr, Srikumaran U (2021). Optimizing fixation for distal biceps tendon repairs: a systematic review and meta-regression of cadaveric biomechanical testing. Am J Sports Med.

[20] Camp CL, Voleti PB, Corpus KT, Dines JS (2016). Single-incision technique for repair of distal biceps tendon avulsions with intramedullary cortical button. Arthrosc Tech.

